# Metagenomic data reveals microbiome characteristics of culture-negative brain abscess samples

**DOI:** 10.1016/j.dib.2023.108893

**Published:** 2023-01-11

**Authors:** Daisy Vanitha John, Meera Purushottam

**Affiliations:** a Department of Neuromicrobiology, National Institute of Mental Health and Neurosciences (NIMHANS), Bangalore, Karnataka, India; b Molecular Genetics Laboratory, Department of Psychiatry, National Institute of Mental Health and Neurosciences (NIMHANS), Bangalore, Karnataka, India

**Keywords:** Culture-negative brain abscess, 16S rRNA PCR, Metagenomics sequencing, Bioinformatics

## Abstract

A brain abscess is a focal collection of pus in the brain parenchyma surrounded by a well-vascularized collagenous capsule in response to an infection. The microbiome of brain abscesses has been shown to be polymicrobial, dominated by uncultivable and anaerobic organisms of odontogenic origin. The data provided in this article includes the sequences of bacterial 16S rRNA gene from three culture-negative brain abscess samples suspected to have poly-microbial aetiology based on Sanger sequencing. DNA was extracted from brain abscess samples, and targeted-metagenomics sequencing was done by amplifying the full-length bacterial 16S rRNA followed by a nested PCR for V3-V4 regions using universal and specific primers. The barcoded amplicons were sequenced on Illumina MiSeq V2 instrument to generate 0.5M, 250bp paired-end reads/sample. The total sequencing reads were 455966, 345746, and 438658 for samples P32, P49, and P8, respectively. Bioinformatics tools such as FLASH, VSEARCH, and QIIME1 were used to process the reads generated for Operational Taxonomic Unit analysis (OTU). Bacterial species belonging to phyla Firmicutes, Bacteroidetes, and Fusobacteria were abundant in samples P49 and P8, which are mainly anaerobic and microaerophilic bacteria. These are typical of the human oral/gut microbiota and are implicated in brain abscess formation. Sample P32 showed the abundance of bacterial species belonging to phyla Proteobacteria and Actinobacteria, which are commonly found in the environment. Raw data files are available at the Sequence Read Archive (SRA), National Center for Biotechnology Information (NCBI), and data information can be found at the BioProject, PRJNA785100 under the accession numbers SRX13271109, SRX13271110, SRX13295897. The data shows the microbiome constitution, including several anaerobic and unculturable bacterial species from culture-negative brain abscess samples. This dataset will be useful for future research on comparative genomics and management of patients with culture-negative brain abscesses.

**Specifications Table**

**Table:** 

Subject	Biology
Specific subject area	Metagenomics, Bacteriology
Type of data	Table, figure, fastq files
How the data were acquired	The 16S rRNA metagenomic sequencing was conducted on Illumina MiSeq V2 paired-end platform and relative abundance of Operational Taxonomic Units (OTUs) was performed using QIIME 1
Data format	Raw data
Description of data collection	The sequenced reads were stitched using FLASH program and chimera were removed using VSEARCH program. Sequences with >97% similarity were grouped under the same OTUs and classified using RDP classifier against Silva database 132 at 97% similarity.The taxonomy classification and relative abundance was done using QIIME1
Data source location	National Institute of Mental Health and Neurosciences Bangalore, Karnataka, India
Data accessibility	The raw sequencing data are available at the Sequence Read Archive (SRA), NCBI (https://www.ncbi.nlm.nih.gov/sra/?term=PRJNA785100), under the BioProject number PRJNA785100. The figure data is available at https://data.mendeley.com/datasets/7rptdf44zt/1
Related research article	D.V. John, B. Aryalakshmi, H. Deora, M. Purushottam, R. Raju, A. Mahadevan, M. B. Rao, S. A. Patil. Identification of microbial agents in culture-negative brain abscess samples by 16S/18S rRNA gene PCR and sequencing. Trop Biomed. 39(4) (2022), pp. 489-498. https://doi.org/10.47665/tb.39.4.002

## Value of the Data

• This data provides the microbiome composition of culture-negative brain abscess samples
• This data shows the presence of several unculturable bacteria in brain abscess samples that cannot be cultured by conventional microbiological method
• The level of bacterial diversity in the microbiome of brain abscess will help to determine the level of disease severity
• This data will be helpful in determining the source of infection
• This data will help to formulate better empiric treatment for patients with culture-negative brain abscesses
• This data can be used to compare microbiome characteristics of brain abscesses of patients in other parts of the world

## 1 Objective

To identify and characterize the microbiome of culture-negative brain abscess samples by metagenomic sequencing. Methods and results are described in detail here, which will benefit the readers of our research article [1].

## 2 Data Description

**Fig. 1:**
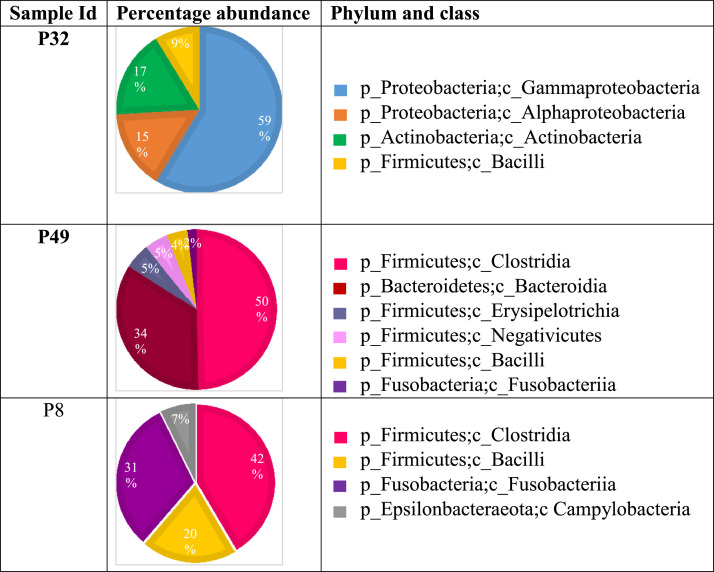
Metagenomics sequencing shows microbiome differences in brain abscess samples. The pie charts show phylum and class indicated in different colours in each sample with percentage relative abundance.

The data reported here are the sequence information and microbiome characteristics of three culture-negative brain abscess samples. The total sequencing reads were 455966, 345746, and 438658 for samples P32, P49, and P8, respectively; after de-replication and chimera removal, the valid reads were 447822, 336437, and 413709, respectively. The processed sequences were used for Operational Taxonomic Units (OTU) analysis, and sequences that possessed more than 97% similarity were grouped into the same OTUs (Fig. 1). Based on the OTU data, the dominant bacterial species in high relative abundance in these samples are listed in Table 1.

**Table 1:** Summary of Sequence information for Bio Project PRJNA785100.

Sample Id	Bio Sample No.	SRA No.	Bacteria identified- sorted by decreasing abundance
P32	SAMN23530455	SRX13271109	g_Vulcaniibacterium;s_uncultured-bacterium s_*Pseudomonas stutzeri* f_Burkholderiaceae;Other;Other s_*Actinomyces viscosus* g_Streptococcus;Other f_Sphingomonadaceae;Other;Other; g_Sphingomonas;Other g_Actinomyces;Other g_Enhydrobacter;s_uncultured-bacterium g_Enhydrobacter;Other g_Cupriavidus;Other

P49	SAMN23530456	SRX13271110	g_Peptostreptococcus;s_uncultured bacterium s_Bacteroidales-oral-clone-MCE7_164 s_Porphyromonas-sp.-HMSC077F02 g_Porphyromonas;s_unidentified g_Parvimonas;Other g_Erysipelotrichaceae-UCG-007;s_uncultured-bacterium s_*Dialister pneumosintes* s_*Bacteroides fragilis* g_Peptoniphilus;s_Peptoniphilus-sp.-KHD5 g_Bacteroides;s_Bacteroides-sp.-HPS0048 g_Streptococcus;s_uncultured-Streptococcus-sp. g_Streptococcus;Other g_Odoribacter;Other g_Fusobacterium;s_uncultured-bacterium

P8	SAMN23530457	SRX13295897	g_Parvimonas;Other g_Streptococcus;other g_Fusobacterium;s_uncultured-bacterium g_Fusobacterium;Other g_Campylobacter;Other

## 3 Experimental Design, Materials and Methods

Three culture-negative brain abscess samples were chosen for metagenomics sequencing that gave mixed chromatograms in Sangers sequencing [1]. Genomic DNA was extracted from brain abscess samples using Nucleospin Tissue DNA extraction kit (Macherey Nagel, Germany) according to the manufacturer's instruction. All Samples were quantified using Qubit DNA HS Assay (Invitrogen), diluted to 5ng, and amplified for full-length 16S rRNA (∼1500bp) using 16S forward (16SF 5′AGAGTTTGATCCTGGCTCAG'3) and 16S reverse (16SR 5′GGTTACCTTGTTACGACTT'3) universal primers [2]. A positive control (internal metagenomic DNA sample) and no template control were included in a step-up strategy for 35 PCR cycles. The PCR products were checked on an agarose gel and used for further amplifying V3-V4 regions of 16S rRNA using specific V3V4 forward (5′-CCTACGGGNGGCWGCAG'-3) and V3V4 reverse (5′-GACTACHVGGGTATCTAATCC'-3) primers with the positive and no template controls by following a step-up strategy for 35 PCR cycles [3]. The PCR products were loaded on a 1% agarose gel to check for positive amplification (∼460bp). The V3-V4 PCR products were purified using AMPure XP beads (Beckman Coulter) and taken for DNA library preparation using NEBNext Ultra DNA Library Prep Kit (Illumina) [4].

The amplicons were end-repaired and mono-adenylated at 3’ end in a single enzymatic reaction. NEB hairpin-loop adapters were ligated to the DNA fragments in a T4-DNA ligase-based reaction. Following ligation, the loop containing Uracil was linearized using USER Enzyme (a combination of UDG and Endo VIII) to make it available as a substrate for PCR-based indexing in the next step. During PCR, barcodes were incorporated using unique primers for each sample. The DNA libraries were checked for fragment distribution on Fragment Analyzer using HS NGS Fragment Kit (1-6000bp) (Agilent). The obtained libraries were pooled and diluted to the final optimal loading concentration. The pooled libraries were then loaded on to Illumina MiSeq V2 instrument to generate 0.5M, 250bp Paired-end reads/sample [5].

Quality check on the raw fastq files was done using FASTQC toolkit to check for base quality, composition, and GC content. Low-quality sequence reads were excluded from the analysis. Forward and reverse reads were stitched together to form long reads greater than 400 bp using the FLASH program with a minimum overlap of 30bp and maximum overlap of 250bp [6]. De-replication was performed using derep_fulllength in VSEARCH for the identification of unique sequences. Chimeric sequences were filtered out using the uchime utility from VSEARCH using a de novo approach [7]. Sequences having a similarity of 97% were grouped together using a *closed_reference* method under a single operational taxonomic unit (OTU) for classification of the whole data. Any OTU that has a count of 1 in a single sample (identified as singletons) were filtered out as these may have been created as experimental artifact. One representative sequence from each OTU was picked up and classified using Ribosomal Database Project (RDP) classifier against Silva database 132 at 97% similarity [8]. Taxonomy classification was done at phylum, order, family, genus, and species level and relative abundance were done using QIIME 1 [9].

## Ethics Statements

Metagenomic sequencing was done for archived samples and informed consent has been obtained from patients for the clinical services. The study was approved by the Institutional Ethical Committee, NIMHANS. (No. NIMHANS/IEC (BS& NS DIV.)/2018 dated 29.5.2018

## CRediT Author statement

**Daisy Vanitha John:** Conceptualization, Methodology, Manuscript preparation; **Meera Purushottam:** Technical advice, Manuscript reviewing and editing.

## Declaration of Competing Interest

The authors declare that they have no known competing financial interests or personal relationships that could have appeared to influence the work reported in this paper.

## Data Availability

Bacteria from brain abscess Metagenome-Bio project (Original data) (Sequence Read Archive (SRA), NCBI).
